# Brisk heart rate and EEG changes during execution and withholding of cue-paced foot motor imagery

**DOI:** 10.3389/fnhum.2013.00379

**Published:** 2013-07-30

**Authors:** Gert Pfurtscheller, Teodoro Solis-Escalante, Robert J. Barry, Daniela S. Klobassa, Christa Neuper, Gernot R. Müller-Putz

**Affiliations:** ^1^Faculty of Computer Sciences, Laboratory of Brain-Computer Interfaced, Institute for Knowledge Discovery, Graz University of TechnologyGraz, Austria; ^2^Faculty of Social Sciences, School of Psychology, Brain & Behavior Research Institute, University of WollongongWollongong, Australia; ^3^Department of Psychology, Neuropsychology, University of Graz AustriaGraz, Austria

**Keywords:** motor imagery, beta ERD, HR response, orienting reflex

## Abstract

Cue-paced motor imagery (MI) is a frequently used mental strategy to realize a Brain-Computer Interface (BCI). Recently it has been reported that two MI tasks can be separated with a high accuracy within the first second after cue presentation onset. To investigate this phenomenon in detail we studied the dynamics of motor cortex beta oscillations in EEG and the changes in heart rate (HR) during visual cue-paced foot MI using a go (execution of imagery) vs. nogo (withholding of imagery) paradigm in 16 healthy subjects. Both execution and withholding of MI resulted in a brisk centrally localized beta event-related desynchronization (ERD) with a maximum at ~400 ms and a concomitant HR deceleration. We found that response patterns within the first second after stimulation differed between conditions. The ERD was significantly larger in go as compared to nogo. In contrast the HR deceleration was somewhat smaller and followed by an acceleration in go as compared to nogo. These findings suggest that the early beta ERD reflects visually induced preparatory activity in motor cortex networks. Both the early beta ERD and the HR deceleration are the result of automatic operating processes that are likely part of the orienting reflex (OR). Of interest, however, is that the preparatory cortical activity is strengthened and the HR modulated already within the first second after stimulation during the execution of MI. The subtraction of the HR time course of the nogo from the go condition revealed a slight HR acceleration in the first seconds most likely due to the increased mental effort associated with the imagery process.

## Introduction

Cue-paced motor imagery (MI) is one of the most frequently used mental strategies in brain-computer interface (BCI) applications (Pfurtscheller and Neuper, 2001; Wolpaw et al., 2002; Faller et al., 2012) for severely handicapped patients (Neuper et al., 2003; Müller-Putz and Pfurtscheller, 2008; Pichiorri et al., 2011) and rehabilitation after stroke (Birbaumer et al., 2008; Kaiser et al., 2012). The major benefit of such a strategy is that the subject has to pay no attention to any externally presented stimuli during self-paced operation at free will. A prerequisite for a good performance, however, is that the subject experience some cue-paced training sessions without or with feedback. Such training consists of a cue stimulus presentation in slightly varying intervals of a few seconds (Pfurtscheller et al., 2008b; Fazli et al., 2012). The location, color or the form of cue indicates either the type of MI (e.g., right vs. left hand) or whether MI has to be performed or withheld (Solis-Escalante et al., 2012). This procedure results in a number of cortical and subcortical processing steps starting with the stimulus perception and decision making and ending with the requested execution of the MI task, whereby the user focuses attention to visually presented cue stimuli. Recently it has been reported that visually cued imagination of left/right hand revealed the highest classification accuracy ~1 s after cue-onset (Fazli et al., 2012). A similar early classification peak during discrimination between visually cued hand and foot MI was reported by Pfurtscheller et al. (2008b). Remarkable in these studies is not only that the highest classification accuracy was found so early after visual stimulation, but also that this early classification peak was observed in nearly every subject. This stability of the early MI classification within and between subjects is of interest and needs further investigation not only in the cerebral (EEG) but also in the autonomic system (heart rate, HR), because both systems are closely linked together. The first who emphasized the close interaction between brain and heart was Claude Bernard (1867), where he especially pointed to the mutual interaction between the two most important body organs (Darwin, 1872/1999; pp. 71–72). Recently, Thayer and Lane (2009) made an extensive review on the cortical control of cardiac activity and about the pathways by which the prefrontal cortex might influence the control of HR. The prefrontal cortex plays a dominant role in the temporal organization of action (Fuster et al., 2000; Haggard, 2005; Soon et al., 2008) whether physically executed or imagined.

Each externally presented stimulus results in a complex total body response, the orienting reflex (OR), first described by Pavlov in 1910 (see Pavlov, 1927) and developed in terms of psychophysiological measures by Sokolov (1960). The OR is an organismic reflex resulting in a range of changes (for details see Barry, 2006) such as EEG alpha desynchronization, cephalic vasodilatation, short-lasting HR deceleration and vasoconstriction in the periphery. The basis of this short-lasting HR deceleration over some seconds was identified in the brief prolongation of the cardiac cycle following stimulation reported by Lacey and Lacey (1978, 1980). Barry (1983) noted that the Laceys' vagally-mediated “primary bradycardia” was the beginning of the HR deceleration observed in the OR context, and suggested that this HR deceleration is an obligatory response marking the initial detection of the stimulus onset transient. Another response to external stimuli is the brisk, short-lasting (~500 ms) beta event-related desynchronization (ERD; Pfurtscheller and Lopes da Silva, 1999) after action-coded visual stimuli - a frequently reported phenomenon (Doyle et al., 2005; Pfurtscheller et al., 2008b; Zhang et al., 2008; Tzagarakis et al., 2010; Wang et al., 2010; Solis-Escalante et al., 2012) related to motor planning, response preparation, response inhibition, and response uncertainty.

The fact that only a minority of people are able to control a BCI properly, without extensive training, depends not only on factors like motivation, mental effort, and mood, but also on automatic operating processes, such as the fast interaction between brain and heart. The aim of this paper is first, to report in detail the brisk reaction of central beta oscillations originating in sensorimotor areas and the HR response after cue-paced movement imagination using a go-nogo paradigm (execution vs. withholding of motor imagery). While the MIgo task was clearly defined, the mental state during the MInogo trials is unknown. Participants were instructed not to move and to relax. Second, this paper aims to discuss how coupled phasic EEG desynchronization and HR deceleration can be interpreted, and provides an understanding of why the classification accuracy in a MI-based BCI can be highest early after the visual cue stimulus. Third, this paper underlines the importance of common EEG and HR changes in BCI research.

## Materials and methods

### Subjects and experimental paradigm

The data of 16 healthy subjects (8 males and 8 females, age 23.6 ± 3.5 years) were recorded. Initially 20 subjects participated in this study; the data from four subjects were discarded because of EEG artifacts and recorded muscle contractions in the EMG during MI. All subjects were seated in a comfortable armchair one meter in front of a computer screen. The computer screen showed cues (duration 2 s) for a go (green circle) and nogo experiment (red circle). The interval between the cues was varied between 11 and 14 s.

The experimental paradigm consisted of two runs with cue-paced motor execution (ME) and three runs with cue-paced MI. The data were recorded in two blocks. In the first block, all participants completed two runs of motor execution. Then, after a pause of about 10 min, all participants completed three runs of MI. For the second block, the participants were instructed to perform kinesthetic MI, i.e., to imagine the sensation of moving their legs in response to the cue. Since none of the participants had previous experience with MI, the first experimental block was included to let the participants pay attention to the kinesthetic aspect of the task. Each run consisted of 40 trials with a go/nogo class probability of 50%. The participants' task was to execute or imagine a brisk movement (dorsiflexion) of both feet following a green circle (MEgo, MIgo), or to withhold the motor task (MEnogo, MInogo) if a red circle appeared.

The order of the blocks did not affect the “baseline” activity of the sensorimotor cortex, meaning that motor execution did not pre-activate the cortex, affecting the estimation of relative changes (ERD/ERS). Evidence against a possible effect of the block order comes from an analysis described in Solis-Escalante et al. (2012), in which the baseline activity preceding nogo trials in the conditions ME vs. MI was compared. No statistically significant differences were found. For further details, e.g., the frequency bands under analysis, see Solis-Escalante et al. (2012).

In this work only data from the MI sessions are reported. The protocol was approved by the Ethics committee of the Medical University of Graz and the subjects gave informed written consent before the experiments.

### Signal recording

The EEG was recorded with fifteen Ag-AgCl electrodes, arranged in three Laplacian derivations around electrode positions C3, Cz, and C4; overlaying the left hand, foot, and right hand sensorimotor cortex. Inter-electrode distance was 2.5 cm. Reference and ground electrodes were attached to the left and right mastoid, respectively. In addition to the EEG, the EMG was recorded from the tibialis anterior muscles in both legs using bipolar derivations. The ground electrode for EMG was attached to the right hip. The EMG was pre-processed before recording. Raw EMG signals were band-pass filtered between 1–1000 Hz, baseline-corrected, full-wave rectified, and integrated with a time constant of 100 ms. EEG and the integrated EMG were recorded with a biosignal amplifier (g.BSamp, Guger Technologies, Austria). Sampling rate was 250 Hz, with filters set between 0.5 and 100 Hz, and a notch filter (50 Hz), for both EEG and integrated EMG. The electrocardiogram (ECG) was recorded from a single bipolar derivation. The negative lead was attached to the chest at the left (mid) clavicular line and the 2nd intercostal space, and the positive lead was attached to the chest at the left midaxilar line and the 6th intercostal space. The ground electrode was placed on the right hip. Self-adhesive Ag-AgCl electrodes were used for these recordings. ECG was recorded with a band-pass filter between 2 and 100 Hz and also sampled at 250 Hz.

### EEG processing

The monopolar EEG data recorded with respect to the left mastoid were transformed with a Laplacian filter to improve the SNR of the signal. The inter-trial variance method (Pfurtscheller and Lopes da Silva, 1999) was used for quantification of the event-related (de)synchronization (ERD/ERS). Time-frequency representations were calculated to visualize the ERD/ERS patterns; only significant ERD/ERS values are displayed (Graimann et al., 2002). ERD/ERS quantification and visualization was managed by the BioSig toolbox (Schlögl and Brunner, 2008; available online http://biosig.sourceforge.net/).

The data were analyzed between 8 and 45 Hz in intervals of 2 Hz. Trials were filtered with a 5th order Butterworth filter, and the ensemble average was subtracted from individual trials. This operation reduces the contribution of phase-locked responses to the ERD/ERS quantification (Kalcher and Pfurtscheller, 1995). The trials were squared and averaged. A moving average window of 250 ms smoothed the ERD/ERS estimation. Significance intervals (*p* = 0.05) were computed with a Box-Cox transformation (Box and Cox, 1964). The reference interval for the relative power changes was 2–4 s prior to cue-onset. For the determination of the most reactive subject-specific beta bands we used the ERD/ERS maps computed from the motor execution task (MEgo) and the discriminability (Cohen's kappa) calculation (Cohen, 1960) between the beta rebound and the reference interval. The beta rebound is much larger during physical execution of movement compared to imagination of the same movement (Solis-Escalante et al., 2012). Hence, to obtain a reliable estimation of the beta band, we used the MEgo trials instead of the MIgo trials. User-specific bands were defined according to the strongest ERS response following the movement execution in MEgo trials. Details on selection of a user-specific band are given elsewhere (Solis-Escalante et al., 2012).

### Calculation of the heart rate (HR) changes

The first step in ECG processing is to detect the QRS complexes in the ECG signal. The interval between consecutive QRS-complexes marks the time from one heart contraction to the next (RR interval; RRI). The QRS-complex was detected automatically based on an algorithm using a filter bank to decompose the ECG signal into various sub-bands (Afonso et al., 1999; implemented in the Biosig toolbox). After detecting every QRS-complex, the RRI is estimated from every pair of successive complexes. The HR value during an RRI equals the inverse of the RRI concerned, and (in beats per minute) is linearly interpolated between two complexes; the resultant time series is averaged across trials from the same class. To obtain HR changes the individual HR signals were synchronously averaged relative to cue-onset (Pfurtscheller et al., 2007).

### Artifact rejection

Trials were rejected if the EMG from the left or right leg exceeded a threshold. For MInogo trials, this threshold was equal to the mean plus three times the standard deviation of the EMG at rest. For MIgo trials, the threshold was equal to the mean plus five times the standard deviation of the EMG at rest, since imagined movements can increase the tonus of the target muscles (Guillot et al., 2007). The “EMG at rest” period was defined as a 5 s interval before cue onset.

After computing the HR, additional trials were rejected by identification of extreme HR changes, i.e., outliers. The first derivative of the HR from each trial was computed, and the absolute value was analyzed using descriptive statistics. Trials were rejected in an iterative fashion: (i) compute the first and third quartile and the interquartile distance from all trials available (class-wise, e.g., separately for MIgo and MInogo); (ii) reject trials if any value of the HR's first derivative was: (1) less than the first quartile minus 1.5 times the interquartile distance, or (2) greater than the third quartile plus 1.5 times the interquartile distance; (iii) repeat until all outliers have been removed.

### Statistical analysis

The statistical significance of EEG band power changes (ERD, ERS) and HR changes was evaluated with a t-percentile bootstrap algorithm, using the implementation of the BioSig toolbox. Upper and lower confidence intervals were obtained from 500 resample repetitions, at confidence levels of 0.05 and 0.01.

Statistical analysis comprised a paired *t*-test for evaluating differences in beta ERD and HR between MIgo and MInogo and a Pearson's product moment correlation for evaluating possible relations between beta ERD and HR deceleration. Comparisons between MIgo and MInogo were analyzed for significant differences with a t-percentile bootstrap algorithm (as described above) for every sample in the time series.

## Results

### EEG time-frequency maps during go and nogo conditions

**Figure 1:**
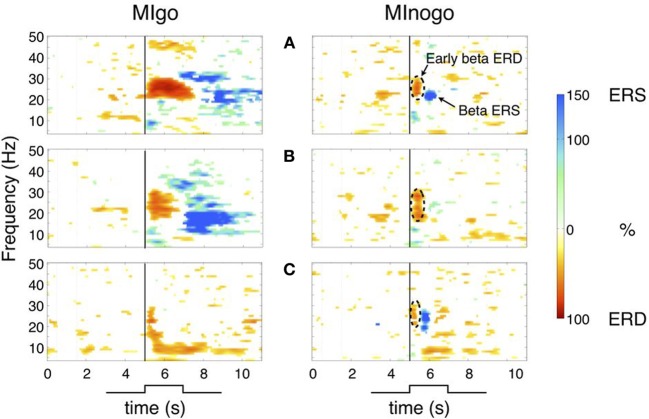
**Time-frequency maps for MIgo and MInogo from three characteristic subjects (A–C) illustrating different reactivity patterns during execution and withholding of MI.** Common for all three subjects is only the early beta ERD during MInogo (marked by stippled circles). Significant ERD values are displayed in “red” and significant ERS values in “blue.” The vertical line at second 5 indicates cue-onset. **(A)** Early beta ERD and beta ERS during MIgo and MInogo. **(B)** Early beta ERD and beta ERS during MIgo, and early ERD and weak beta ERS during MInogo. **(C)** Early beta ERD during MIgo and early beta ERD with beta ERS during MInogo.

To obtain an overview of the dynamics of sensorimotor rhythms in the go (MI) and nogo condition (withholding of MI), time-frequency maps were calculated separately for each condition. Examples from three characteristic subjects (A–C) are shown in Figure 1. The maps in Figures 1A,B (left panels) display the classical response in the beta band during imagined dorsiflexions of both feet (MIgo) namely a long-lasting (~1–2 s), midcentrally localized, peri-imagery ERD during execution of the imagery process and a post-imagery ERS (beta rebound) after termination of this process. But subjects can also show an early beta ERD without following beta ERS during MIgo (example see Figure 1C, left panel), similar to the early ERD in the nogo condition (Figures 1A–C, right panels). Because of the variable duration of each imagery task and the alignment of the averaging process to cue-onset and not to the exact end of MI, the beta ERS (beta power increase) in the go condition is relatively long-lasting. In the nogo condition (Figure 1 right panels) the early beta ERD starts immediately after cue-onset, has a duration of ~500 ms and is terminated by a brisk beta ERS with a maximum ~1 s after cue-onset (panels A–C).

The subject-specific beta band (mean ± *SD*) determined from the time-frequency maps and used for further analyses was between 18.8 ± 4.7 and 28.4 ± 4.8 Hz. The magnitudes and latencies of beta power minima (largest ERD magnitude) and HR minima measured within the first second after stimulation onset are summarized in Table 1 for both the go and nogo conditions.

**Table 1:** **Mean ± *SD* (16 subjects) for latency (ms) and magnitude (%) of early beta power minimum (beta ERD) and change of HR for go and no conditions**.

	**ERD**				**HR**			
	**Latency (ms)**		**Amplitude (%)**		**Latency (ms)**		**Amplitude (%)**	
	**Mean**	***SD***	**Mean**	***SD***	**Mean**	***SD***	**Mean**	***SD***
MI go	471	211	−39.82^*^	20.56	640	224	−1.00	1.84
MI nogo	397	139	−32.00^*^	23.78	652	276	−1.94^*^	1.28

**Figure 2:**
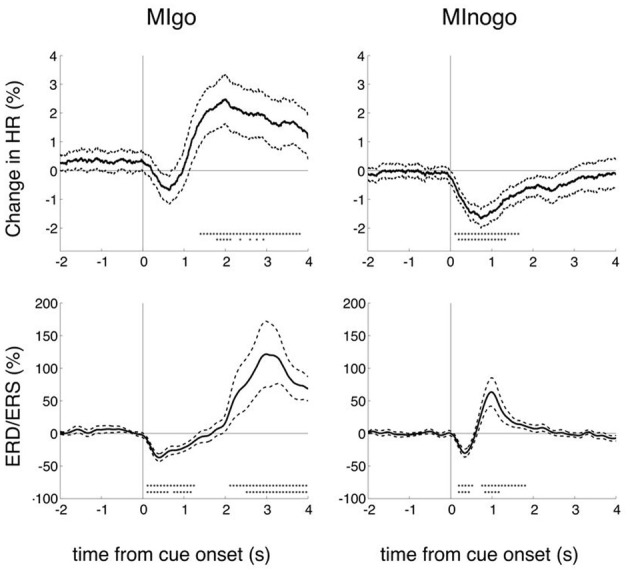
**Grand averages of percentage HR and beta power (ERD/ERS) changes for execution (MIgo) and withholding (MInogo) of foot motor imagery.** In addition to the mean and the standard error (stippled lines) also the significant changes (bootstrap) are displayed (one asterisk vertically *p* < 0.05, and two asterisks vertically *p* < 0.01). Data of 16 subjects. Note the significant HR decrease in the nogo condition and the significant early beta ERD in both the go and nogo conditions.

* indicate statistical significance (p < 0.01, see Figure 2).

The beta ERD was significantly (paired *t*-test) larger (39.82 vs. 32.00%; *t* = 5.37, *p* < 0.001) during MIgo compared to MInogo, but its peak latency was not significantly longer (471 vs. 397 ms; *t* = 1.05, ns). In contrast, HR deceleration was somewhat smaller in MIgo vs. MInogo (1.00 vs. 1.94 %, *t* = 2.03, *p* = 0.06); again, peak latencies (640 vs. 652 ms) did not differ significantly (*t* = 0.18, ns).

### Changes of beta power and HR during execution (go) and withholding (nogo) of motor imagery

Grand averages of changes in percentage HR (upper panels) and EEG beta power (lower panels) for both conditions are displayed in Figure 2. Reference interval is 2–4 s before cue presentation. Beside the mean ± SE also significant changes (bootstrap *p* = 0.01) are indicated. Excepting the HR changes in the go condition, all other HR and EEG changes (HR deceleration and EEG beta desynchronization) were significant in the first second after cue presentation in go and nogo conditions (*p* < 0.01). While in the go condition the HR showed a significant (*p* < 0.01) acceleration after the brisk deceleration, no further significant HR changes were observed after the deceleration in the nogo condition.

**Figure 3:**
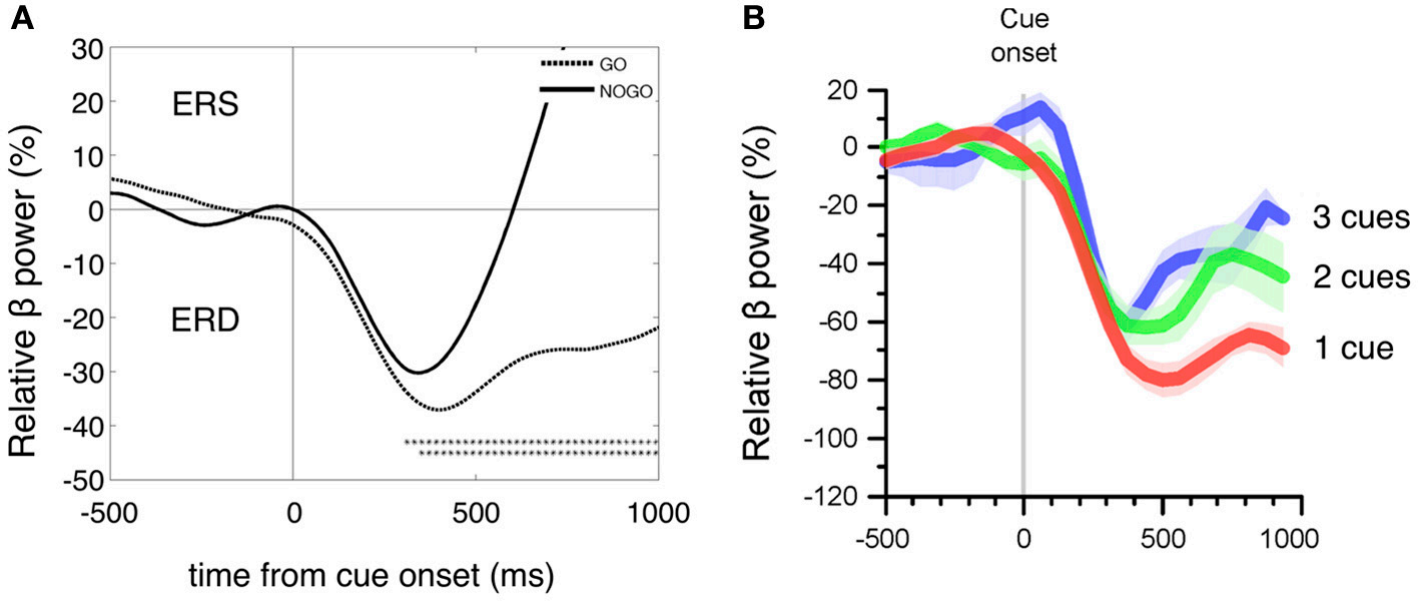
**(A)** Details of the grand average beta power time courses (ERD/ERS) of 16 subjects within the first second after cue onset for MIgo (dashed line) and MInogo (continuous line). Beta power was compared between MI go and MInogo (bootstrap, one asterisk *p* < 0.05, two asterisks *p* < 0.01) and revealed significant differences starting at 250 ms after cue onset. **(B)** Time courses of relative beta power changes after a visually instructed reaching task with one, two, or three possible target directions (with permission modified from Tzagarakis et al., 2010). Note the similar early beta ERD with a maximal magnitude at ~400 ms in EEG **(A)** and MEG **(B)** recordings during different motor tasks.

For a direct comparison of the beta power decrease during motor planning in the go/nogo conditions after visual cue presentation with a similar 248-channel MEG study (Tzagarakis et al., 2010) the reference interval was changed to 0–1 s prior to cue onset [the standard reference interval for the processing of all EEG and HR data (Figure 2) was 2–4 s prior to cue onset in accordance with the work of Solis-Escalante et al. (2012)]. Common for both studies (Figures 3A,B) is the early beta power decrease from cue onset (Tzagarakis et al., 2010 reported a delay of 120 ms) not dependent on the type of information provided by the visual stimulus but rather determined by a constant visuomotor delay. The different behavior of the beta ERD between execution and withholding of MI starts between 200 and 300 ms after cue onset, indicating on the one hand that the cue stimulus-induced early beta ERD peaks at ~400 ms, and on the other hand that the cognitive task (execution of MI) is able to modulate this desychronization.

**Figure 4:**
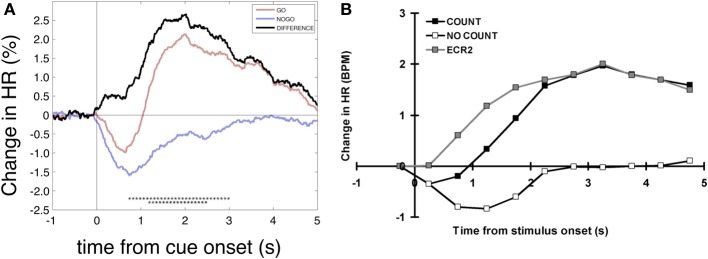
**Heart rate changes after go and nogo cues. (A)** Grand average time courses of percentage HR changes aligned to cue onset referenced to an interval 1–0 s prior to cue onset (different from the text and analyses above). Displayed are the HR responses for MIgo, MInogo and their difference (hypothetical HR acceleration). Significant differences between conditions are marked with black asterisks (bootstrap, one asterisk *p* < 0.05, two asterisks *p* < 0.01). **(B)** Grand average HR response for silent counting of tones (COUNT) vs. no-count (NO COUNT) and hypothetical HR response (DIFFERENCE). Modified from Lawrence and Barry (2010).

When searching for possible interpretations of the HR responses in the go and nogo condition we subtracted the nogo HR response from the go response (MIgo minus MInogo) and achieved a new response pattern termed “hypothetical” HR response (Figure 4A) with a HR acceleration starting immediately at cue onset and a maximum at ~2 s latter. Remarkable is the similarity between the “hypothetical” HR response in Figure 4A and the HR responses in a similar no/go study without any motor preparation displayed in Figure 4B (Lawrence and Barry, 2010). The brisk HR deceleration is the dominant feature in both studies.

### Interdependency between early ERD and brisk HR deceleration

**Figure 5:**
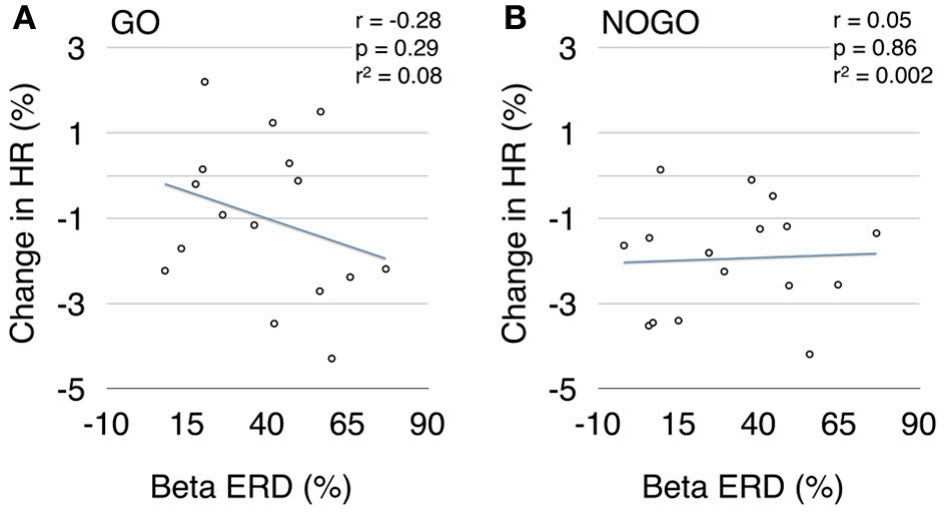
**Scatter plot between early beta ERD (%) and brisk HR decrease (%) during motor imagery (A) and withholding of motor imagery (B)**.

When testing linear inter-subject correlations between beta ERD and HR deceleration within the first second after cue-onset, no significant correlation was found for MInogo (Pearson correlation coefficient *r* = 0.05, *p* = 0.86; see Figure 5B); the correlation for MIgo was in the expected direction (greater deceleration with greater ERD) but failed to reach significance (*r* = −0.28, *p* = 0.29; see Figure 5A).

## Discussion

The following findings need special attention: (i) the slightly different reactivity patterns in the EEG during go and nogo conditions within the first second in the form of an early beta ERD, (ii) the different magnitudes of brisk HR decelerations in both conditions and (iii) that no significant correlation was found between early ERD and HR deceleration.

### EEG changes during execution (go) and withholding (nogo) of MI

In the EEG rhythms, various phenomena have to be differentiated during execution and withholding of MI. First the early beta ERD with a peak ~400 ms after cue-onset observed in both conditions, second the longer-lasting beta ERD associated with the conscious intention of MI (peri-imagery ERD) in the go condition, and third the beta rebound (post-imagery ERS) terminating the beta ERD. In the go condition the peri-imagery ERD becomes either superimposed on the early beta ERD within the first second (see Figures 1A,B) or the early beta ERD remains in the go condition (Figure 1C). Whether the absence of a longer-lasting beta ERD acts as indicator for a not correctly performed imagery task can only be speculated. The ERD represents a process to enable focal attention so that information processing may be optimized (Lopes da Silva, 1991) or, in other words, the ERD indicates a state of cortical activation (Gerloff et al., 1998; Pfurtscheller and Lopes da Silva, 1999; Leocani et al., 2001; Doyle et al., 2005). The beta ERS corresponds to a state of deactivation of the motor cortex circuitry and signifies active immobilization (Salmelin et al., 1995). In the nogo condition the beta ERD begins to recover 400 ms after cue onset and reaches its peak ERS at 1000 ms (see Figure 2 right lower panel and Figures 1A,C). Support for an activation of the foot representation area during MIgo came from fMRI studies. Physical execution but also mental simulation of foot/toe movement is not only accompanied by peri-imagery and mid-centrally localized beta ERD (Pfurtscheller et al., 2006a) but also by a positive fMRI BOLD signal localized to the foot representation area (Ehrsson et al., 2003).

In respect to the early ERD direct recordings from single neurons of the monkey‘s motor cortex are of interest. Such neurons start to discharge 60–80 ms after occurrence of a visual cue, indicating the direction of the upcoming movement (Georgopoulos et al., 1982). Preparatory changes in neuronal activity prior to movement execution have been documented also in monkey premotor and primary cortex 100 ms after the visual cue signal (Riehle and Requin, 1995). These studies show clearly that the motor cortex is engaged as early as about 100 ms after an informative visual cue indicates to execute or withhold a specific motor task. Similarly, in another monkey study, the beta activity displayed an early ERD in go and nogo conditions starting ~110 ms after visual stimulus presentation (Zhang et al., 2008). Therefore, we can conclude that the early phasic beta ERD observed in our data is a sign of preparatory activity in motor cortex networks triggered by the visual stimulus and modulated by the cognitive task.

Most interesting is that the early beta ERD displays a slightly but significant stronger magnitude (*p* < 0.01) and longer peak latency (n.s.) during MIgo than in MInogo (Table 1 and Figure 3A). The decision to execute or withhold MI is estimated to occur ~190 ms after stimulation (Zhang et al., 2008). Support for such an early stimulus induced preparatory activity in motor cortex networks came from two MEG studies (Tzagarakis et al., 2010; Wang et al., 2010). In Wang's study subjects were asked to imagine four different center-out movements using the wrist after visual target onset. The source-space multivariate test revealed that by 300–400 ms after target onset the cortical activity becomes highly modulated and can be used to decode the type of covert movement. Tzagarakis et al. reported on a 248-channel whole head MEG study while subjects performed a visually instructed reaching task with one, two, or three possible directions. The early beta ERD with a maximum at ~400 ms is heightened with decreased directional uncertainty (see Figure 3B). Remarkable is that the early beta ERD displays a peak at 500 ms in such two completely different studies, one with MEG and response uncertainty and the other with EEG and MI. This suggests that the early beta ERD can be maximal around 500 ms after cue onset not only in EEG and MEG recordings, but also can be modulated by different cognitive processes such as MI or response uncertainty.

### HR changes during execution (go) and withholding (nogo) of MI

The initial obligatory post-stimulus HR deceleration in the OR context mentioned in the Introduction is exemplified in response to the nogo cue, as illustrated in the right panel of Figure 2. This brisk HR response is significantly and clearly of larger magnitude when compared with the response during MI (go condition; Figure 2 left). Of interest is that this HR response is nearly identical with the “hypothetical” HR response difference in a similar go-nogo experiment where subjects were required to silently count the number of tones (go condition); in the nogo condition the subjects were told to relax during tone presentation (Lawrence and Barry, 2010; see also Figure 4B). These authors linked their hypothetical HR acceleration to the increased mental effort of the count condition. The hypothetical HR acceleration during MI is linked to the mental effort in the same fashion. A HR acceleration is characteristic for an increased mental effort during the simulation of a motor act (MI) (Decety et al., 1991; Oishi et al., 2000; Pfurtscheller et al., 2006b, 2008a).

### Coupling between early beta ERD and brisk HR deceleration

The beta ERD and concomitant HR deceleration after stimulation is not an isolated phenomenon but is also found during preparation for movement or even in the resting brain. The pre-movement beta ERD and the pre-movement HR deceleration are well documented phenomena (Damen and Brunia, 1987; Papakostopoulos et al., 1990; Florian et al., 1998; Pfurtscheller and Lopes da Silva, 1999). A slow cyclic (in intervals of ~10 s) central beta (alpha) power decrease and a nearly simultaneous HR decrease (beat-to-beat increase) in the resting state was reported recently (Pfurtscheller et al., 2012a,b). If the desynchronization of sensorimotor rhythms prior to movement can be interpreted as a correlate of preparatory activity then also the early beta ERD after cue presentation can be seen as stimulus triggered preparatory activity in the sensorimotor system. This preparatory activity, and also the HR deceleration, very likely result from routine processes operating automatically and unconsciously (Koch, 2003; Haggard, 2005). The EEG-HR coupling prior to voluntary movement, during rest and after visual stimulation can be seen as an example of the fast interaction between brain and heart mediated by pathways most likely from the prefrontal cortex to cardiovascular nuclei in the brain stem and vagally via the sino-atrial (SA) node to the heart (Thayer and Lane, 2009). The SA node responds very quickly to vagal (~150 ms latency) in contrast to sympathetic (~1–2 s) influences (Smyth et al., 1969).

Visual information is not only mediated by the lateral geniculate body, but also by the superior colliculus. The superior colliculus is important for the control of saccadic eye movements (Krauzlis et al., 2004), and is believed to be responsible for a transient increase in blood pressure and HR by drive of sympathetic activity (Iigaya et al., 2012) Electrical stimulation of the optic tract also increases blood pressure and HR, and inhibits baroreflex vagal bradycardia, mediated by the superior colliculus (Cheng et al., 2001). The participation of the superior colliculus in reflexive vagal mediated primary bradycardia (Lacey and Lacey, 1980; Barry, 1986) is therefore very unlikely.

That no significant correlation was found between early beta ERD and HR deceleration during MIgo (see Figure 5A) might be explained by different reasons. While the MIgo cardiac response can be conceptualized as an additional cardiac acceleration reflecting mental effort, superimposed on the reflex bradycardia triggered by the cue (see Figure 4A), a comparative examination of the corresponding beta power response profiles gives a more complex picture (see Figure 3A). Here the reflexive early beta ERD (peaking at ~400 ms) is superimposed on the peri-imagery beta ERD induced by the conscious mental simulation of foot movement. Although the early beta ERD with the following beta ERS in the nogo condition is an automatically induced response, only the early beta ERD can be considered as a component of the OR. The relationship between these differences in the EEG and HR response profiles during MIgo is complex and needs further exploration, possibly going beyond the linear correlation of responses in the short interval tested here. Another reason for the non-significant correlation could be that in all subjects the EEG was recorded at the vertex only (Cz: Laplacian derivation) although it is known from multichannel MEG recordings that the source of the beta band ERD can vary in the peri-Rolandic region across subjects (Tzagarakis et al., 2010). Beside the EEG also the ECG can induce some variability. The HR displays two preferential rhythms, one around 0.25 Hz (respiration) and one around 0.1 Hz (blood pressure or Mayer waves) (De Boer et al., 1985) as well as fluctuations induced by “central commands” (see review Benarroch, 1993; Thayer and Lane, 2009). Slow intrinsic HR fluctuations during rest can be as high as ~10 % of the mean HR (Pfurtscheller et al., 2012a) and mask the relatively small (~2%) brisk HR deceleration induced after stimulation.

## Conclusion

The early beta ERD with the largest magnitude at ~400 ms is slightly, but significantly (*p* < 0.01) larger during execution (go) as compared to withholding of MI (nogo). At the same time the HR displays a significant (*p* < 0.01) brisk deceleration only during withholding of MI. Both the early beta ERD and the HR deceleration are the result of an automatic operating process and probably part of the OR (Barry, 2006). Of interest, however, is that both automatic reactions in brain and heart can be modulated by the increased mental effort associated with execution of MI. The calculation of the “hypothetical” HR acceleration introduced by Barry (1984) offers a new way to explore the mental effort during motor imagery early after stimulation. A high mental effort during imagined movements is accompanied by a HR increase (Decety et al., 1991; Oishi et al., 2000). Such indicator of mental effort could be used in a BCI to improve its performance (Pfurtscheller et al., 2010). For online monitoring of the mental effort in a cue-paced MI task, the averaged HR response of the MInogo condition obtained in a pre-experiment has to be subtracted from each ongoing HR response (calculation of the “hypothetical” HR). By this way the mental effort can be supervised and an intervention made if necessary.

The early beta ERD is an important brain feature that might be responsible for the early classification peak in imagery-based BCIs because of its somatotopic organization as demonstrated recently (Pfurtscheller et al., 2008b). The close relationship between early ERD and action-coded visual stimulation also was discussed elsewhere (Waldert et al., 2008; Wang et al., 2010). If the visual stimulus indicates only one specific type of MI the attention is focused on this specific imagery task and results in an early beta ERD in both conditions (go/nogo). The results suggest that the early classification peak in cue-paced BCIs is very likely the result of an automatic operating process induced by the preparatory visual cue stimulus. This hypothesis, however, needs to be further tested, possibly with an auditory cue stimulus.

The fast interaction between brain and heart is a basic factor in BCI research. HR slowing and concomitant pericentral EEG desynchronization is not only characteristic for the planning and/or preparation of a motor task (e.g., ME or MI), but also a dominant feature immediately after visual cue presentation. Still open for research and discussion is the link between slow (~0.1 Hz) spontaneous HR and EEG oscillations during awake rest (Pfurtscheller et al., 2012a,b). In this respect, the finding that the relation between early beta ERD and brisk HR deceleration is very likely the result of an automatic process, operating after visual cue presentation, is a small step to improve the success rate in BCI research and focus research attention on the importance of intrinsic HR and blood pressure oscillations at a frequency of around 0.1 Hz.

### Conflict of interest statement

The authors declare that the research was conducted in the absence of any commercial or financial relationships that could be construed as a potential conflict of interest.
